# Outcomes of sutured scleral-fixated intraocular lens implantation combined with penetrating keratoplasty

**DOI:** 10.1186/s12886-024-03603-6

**Published:** 2024-08-12

**Authors:** Abdulmohsen Almulhim, Waleed K. Alsarhani, Bader Alanazi, Abdulrahman Alfaleh, Mohanna Aljindan, Rahaf M. Al Malawi, Abdulaziz Al-Somali

**Affiliations:** 1 Department of Ophthalmology, College of Medicine, Jouf University, Sakakah, Al-Jouf, Saudi Arabia; 2 Department of Ophthalmology and Vision Sciences, University of Toronto, 340 College Street, Toronto, ON M5T 3A9 Canada; 3 Department of Ophthalmology, King Faisal Specialist Hospital & Research Centre, Riyadh, Saudi Arabia; 4 Department of Ophthalmology, College of Medicine, Imam Abdulrahman Bin Faisal University, Dammam, Saudi Arabia; 5 College of Computer and Information Sciences, Princess Nourah Bint Abdulrahman University, Riyadh, Saudi Arabia; 6 Department of Ophthalmology, College of Medicine, King Faisal University, Al-Ahsa, Saudi Arabia

**Keywords:** *Scleral-fixated lens implant*, *Keratoplasty*, *Corneal transplant*

## Abstract

**Background:**

The purpose of the study was to assess visual outcomes, complications, intraocular lens (IOL) stability, and corneal status after sutured scleral-fixated intraocular lens implantation combined with penetrating keratoplasty (PKP).

**Methods:**

This retrospective single-arm cohort study included patients who underwent PKP and sutured scleral-fixated intraocular lens implantation between 2013 and 2018 at the Dhahran Eye Specialty Hospital. The eyes were examined postoperatively at 1, 3, 6, 12, and 24 months. Corneal status, complications, and IOL status were also evaluated periodically, and the number of eyes with a BCVA of > 20/200 was recorded.

**Results:**

Twenty-two eyes from 22 patients were included. The median duration of follow-up was 3 (IQR 1.8; 4.4) years. Reasons for surgery were traumatic globe rupture (six eyes, 27.3%), bullous keratopathy (nine eyes, 40.1%), failed previous graft (five eyes, 22.7%), and corneal scarring (two eyes, 9.1%). Twelve (54.5%) eyes showed a BCVA of > 20/200 (non-blind) at 12 months after surgery and only five (22.7%) before surgery. Twelve months after surgery, 13 patients showed an improvement in BCVA in two lines (59.1%), seven remained the same (31.8%), and 2 deteriorated (9.1%). The indication for surgery (*p* = 0.2) and the stability of the sutured-scleral fixated IOL (*p* = 0.8) were not associated with an improvement in BCVA at the final follow-up. The corneal graft remained clear in nine eyes (40.9%) at a median duration of 3 years. The overall average survival period for all corneal grafts was 42.9 months.

**Conclusions:**

The combination of sutured scleral-fixated intraocular lens implants and PKP is an effective intervention for preserving visual acuity in patients with complex cases. However, the risk of graft failure and then need for repeat transplantation should be taken into consideration.

## Background

Corneal pathologies such as bullous keratopathy and scars in eyes without sufficient capsular support are treated with keratoplasty and scleral-fixated intraocular implants [1, 2]. While the outcome of keratoplasty alone is promising, the underlying cause of corneal pathology determines the success of implantation and survival of graft [3, 4]. Several procedures, including suturing of anterior chamber intraocular lenses (IOLs) and posterior chamber IOL to the iris, sulcus fixation of IOLs, glue-assisted fixation, and scleral-fixation of IOLs, have been described to fixate an IOL in eyes with weak or no lens capsule [5–7]. The latter can involve fixation by suturing the IOL sub-conjunctival or trans-scleral haptic, which includes using a scleral tunnel to bury the suture [8].

The outcomes of scleral-fixated IOL implantation combined with penetrating keratoplasty (PKP) were described as early as 1992 [9]. However, technologies for performing PKP, cataract surgeries, and IOL materials have improved markedly. Such a combined surgery has promising outcomes for one or two years, but the results are of a less-than-desired level in eyes with a history of severe trauma [10]. Several case reports and studies using small samples have been noted in the literature [11–13]. The aim of the present study was to report the visual outcome, complications and IOL stability after combined PKP with sutured scleral-fixated IOL.

## Methods

This study was approved by the Institutional Review Board of the Dhahran Eye Hospital. All patients underwent sutured scleral-fixated IOL implantation combined with PKP between 2013 and 2018 at the Dhahran Eye Specialist Hospital in the Eastern Province of Saudi Arabia included in the study. After delineating their identities, their health records were reviewed. As this was a retrospective cohort study, the requirement for informed consent for surgery was waived. The Dhahran Eye Specialist Hospital Corneal Transplantation Patient Registry was the primary resource for all preoperative, intraoperative, and postoperative patient information.

Demographic information collected from each patient included age, sex, and eye involvement. Data on history of ocular trauma, prior ocular surgery, and glaucoma were also obtained. Visual acuity was measured using a Snellen chart held at a distance of 6 m, with and without a pinhole occluder. The anterior segment of each eye was evaluated using a slit-lamp. The posterior segment was evaluated using an indirect binocular ophthalmoscope. Intra-and postoperative complications were also documented. The eyes were examined at 1, 3, 6, 12, and 24 months postoperatively and at the last follow-up. Data on the best-corrected visual acuity (BCVA), IOL status, status of the transplanted cornea, intraocular pressure, and other complications and their management were collected. The difference between the BCVA at follow-up and before surgery was defined as success. Improvements better than two lines were considered successful. Patients were graded as “non-blind” if BCVA was better than 20/200. Vision worse than 20/200 with perception of light was graded “blind.” An eye with no perception of light is termed “absolutely blind.”15 Main outcome measures were graft survival rate, improvement in BCVA by two lines, intraoperative and postoperative complications, IOL position, and IOL suture status.

### Surgery

Surgeries were performed under general anesthesia. The literature describes the details of PKP and scleral fixation procedures [14]. In most cases, the donor cornea was sutured to the recipient cornea using 16 interrupted sutures. The donor material supplied by the Central Bank in Riyadh was imported, evaluated, preserved, and provided by international agencies. In the present study, TYPE 66 IOL (Morcher, Germany) single-piece, all-polymethylmethacrylate (PMMA) biconvex lens with an overall length of 13 mm, optic size of 6.5 mm, and suture eyelets at the apices of the haptics were used.

The steps involved in IOL implantation are summarized as follows: A conjunctival peritomy and either a scleral groove or a lamellar scleral flap were made at the site of the intended suture fixation. After trephining the host cornea button, the problematic IOL (if present) was explanted, and the posterior chamber IOL (PCIOL) was implanted using an open-sky approach. In many cases, anterior vitrectomy has been performed using an automated instrument with a fiber-optic light pipe to illuminate the vitreous. Thorough anterior vitrectomy is necessary to prevent anterior vitreous prolapse around the IOL optic or through peripheral iridectomy sites, vitreous-induced displacement of the IOL, or vitreous incarceration into the scleral suture tracks. Iris adhesions were lysed whenever warranted, and large iris defects were repaired using 10 − 0 poly-propylene suture iridoplasty. CIF-4 needle 1516 (Ethicon Inc., Somerville, NJ, USA) with a 20-cm, double-armed 10–0 polypropylene (Prolene) suture was used for transscleral PCIOL fixation. This needle is longer and less cumbersome than short needles such as the TG-140 microneedle, designed for transscleral fixation via a limbal, cataract-style wound. The open-sky approach facilitates access to the ciliary sulcus using a CIF-4 needle 1516. The polypropylene suture was tied to the apex of the PCIOL haptic using either a girth hitch, necessitating a double-ended suture, or a single-ended stitch. The sutures were passed outward through the ciliary sulcus and sclera at around 0.8–0.9 mm posterior to the limbus. The PCIOL was positioned in the posterior chamber with haptics in the sulcus and placed in an oblique meridian (usually 2:00 and 8:00) to avoid the involvement of the long posterior ciliary arteries and nerves. After positioning the PCIOL, the sutures were tightened before tying. With the girth hitch, the double-ended sutures were tied in a mattress-like fashion. Using the single-stitch method, a single suture was tied onto itself after taking a second partial-thickness pass through the sclera. The knots were covered with conjunctival flaps or lamellar partial-thickness scleral flaps.

### Statistical analysis

Statistical analysis was performed using the Statistical Package for Social Sciences (SPSS) 28.0 software (IBM Inc., Chicago, IL). Categorical variables were presented in frequencies and percentages, and continuous variables were presented as means ± standard deviation. The Chi-squared test was used to examine the differences between categorical variables. Kaplan-Meier survival plot was used to illustrate overall survival period for all included grafts. All results were considered to be statistically significant at *P* < 0.05.

## Results

The study cohort included 22 eyes (22 patients) (Table 1). The median age was 59.2 years (IQR 45.2; 74.2). Seventeen male (77.3%) patients and 10 (45.5%) right eyes were included. Reasons for surgery were traumatic globe rupture (six eyes, 27.3%), bullous keratopathy (nine eyes, 40.1%), failed previous graft (five eyes, 22.7%), and corneal scarring (two eyes, 9.1%). The eyes included in the cohort had corneal neovascularization (seven eyes, 31.8%), glaucoma (six eyes, 27.3%), vitreous hemorrhage (two eyes, 9.1%), retinal pathologies (four eyes, 18.2%), Ahmed glaucoma drainage implant (two eyes, 9.1%), other comorbidities (post-vitrectomy) (two eyes, 9.1%). In eight eyes, no other ocular comorbidities were documented in the eight eyes. At presentation, eight eyes had IOLs implanted in the anterior chamber, two had IOLs in the posterior chamber, five were aphakic, and seven had traumatic cataracts with zonular instability necessitating secondary IOL implantation. The mean duration of surgery was 114 ± 23 min. The median follow-up duration was 3 years (IQR 1.8; 4.4).

**Fig. 1:**
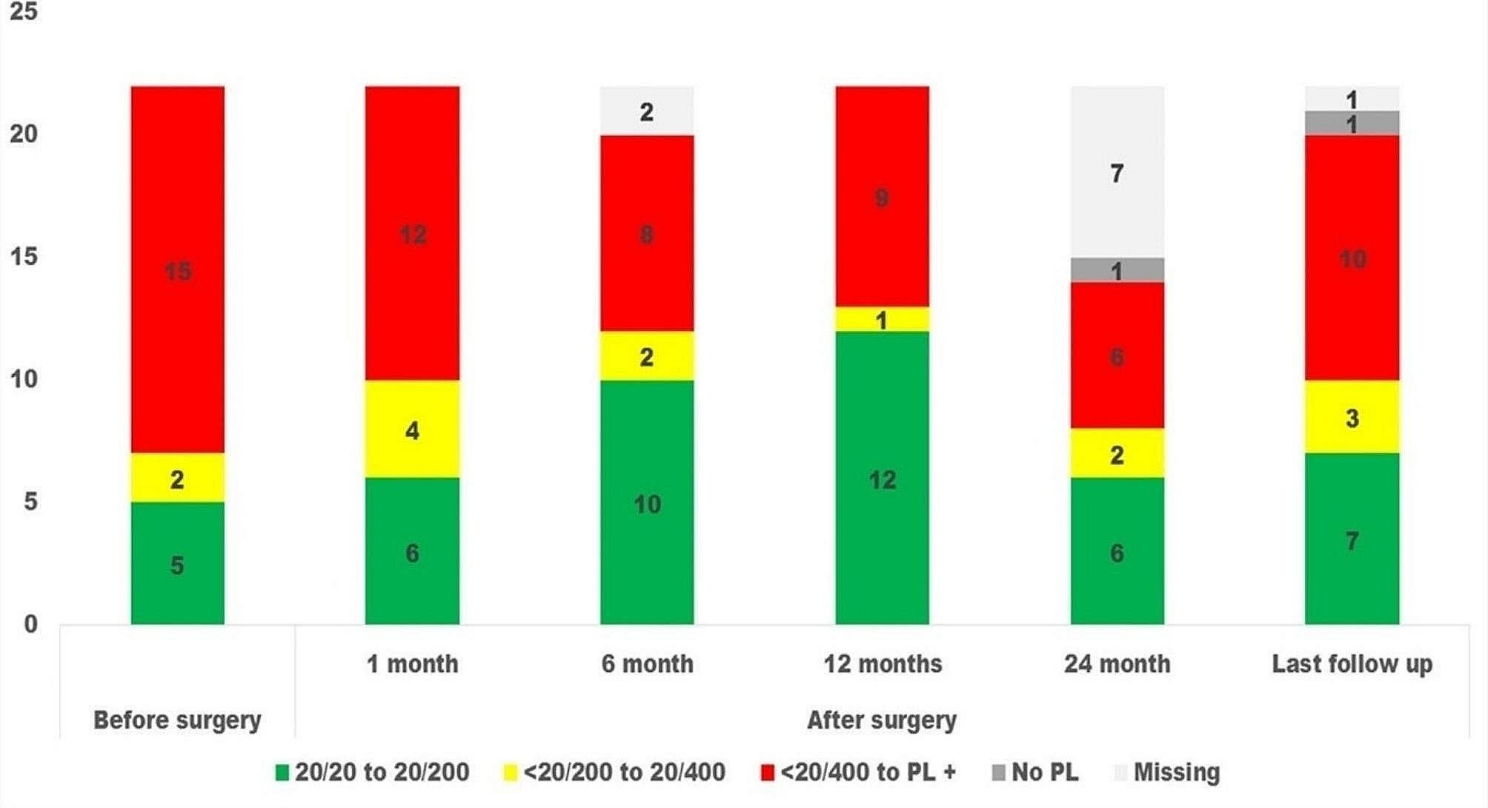
Visual impairment grades before and at different follow-ups after penetrating keratoplasty and scleral fixated intraocular lens implant surgery

Twelve (54.5%) eyes showed a BCVA of > 20/200 (non-blind) at 12 months after surgery compared to only five (22.7%) before surgery. The BCVA before and at different time points after surgery are shown in Fig. 1. At 12 months after surgery, BCVA improved by two lines in 13 patients (59.1%), remained the same in seven patients (31.8%), and deteriorated in two patients (9.1%) relative to BCVA before surgery. Improvement in vision by two lines was noted in six of the 10 eyes examined after three years. The corneal graft was clear in nine eyes (40.9%) at a median duration of 3 years after SSF combined with PKP. Graft failure occurred, on average, at 14.70 ± 10.55 months.

No intraoperative complications were observed. Among the 16 eyes (72.7%) that did not have glaucoma preoperatively, new onset glaucoma was seen in 5 eyes (31.25%) at a median duration of 3 years postoperatively; four eyes controlled by medications, and only one eye required glaucoma surgery. No complications were observed. Only three eyes (13.6%) had unstable intraocular lenses at a median duration of three years. The first patient had an IOL tilt; however, vision did not improve with refraction and was most likely poor due to the patient’s uncontrolled glaucoma and corneal graft failure. The second patient had an IOL tilt, which did not affect his vision. The third patient had a subluxated IOL with no improvement in vision with refraction and had coexisting uncontrolled glaucoma and advanced cupping.

**Fig. 2:**
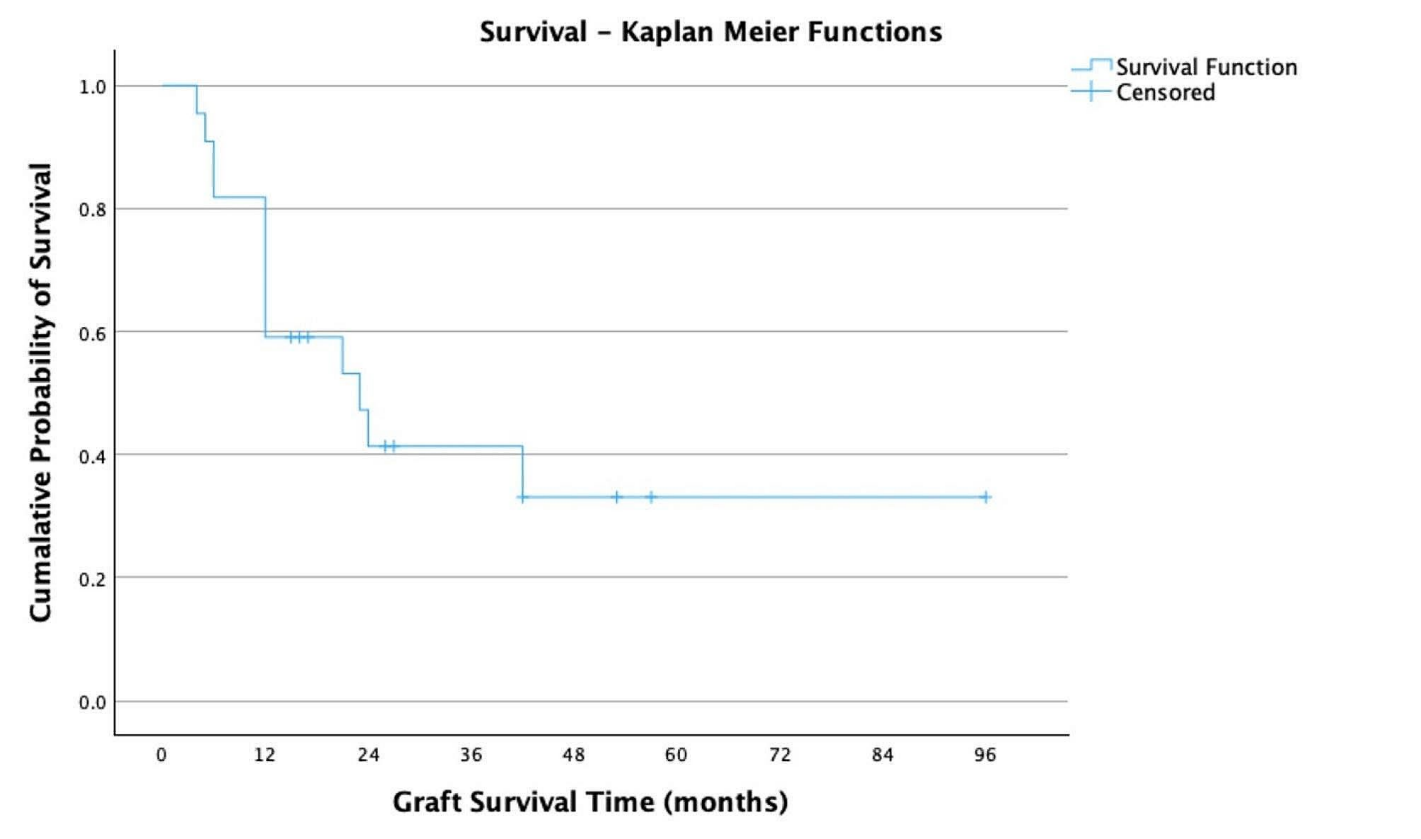
Kaplan-Meier survival plot for all included cases, showing survival probabilities over time

Table 1 demonstrates the clinical characteristics of all included patients. The factors related to vision improvement in the two lines at 12 months after surgery are shown in Table  2. The type of comorbidity (*p* = 0.5) and indication for surgery (*p* = 0.2) were not significantly associated with the two-line improvement in vision 12 months after surgery. The stability of the scleral-fixated IOL was also not associated with a two-line improvement in BCVA at 1-year follow-up. The overall average survival period for all corneal grafts was 42.9 months (95% confidence interval [CI]: 25.3 to 60.6). The Kaplan-Meier survival plot is demonstrated in Fig. 2.

**Table 1:** Clinical characteristics of included patients

Serial number	Age range	Gender	Eye	Preop VA	Preop IOP	Postop VA at last visit	Lens	Indication	Graft status at last FU	IOL status at last FU
1	65–70	M	R	20/200	14	20/125	Pseudophakic	ACIOL, BK	Clear	Stable
2	65–70	F	R	20/400	15	20/400	Aphakic	BK	Clear	Stable
3	60–65	M	L	HM	28	HM	Aphakic	Graft failure	Failed	Tilted
4	35–40	M	L	20/100	19	20/30	Pseudophakic	BK, ACIOL	Clear	Stable
5	25–30	M	R	CF	16	20/50	Phakic	Trauma	Clear	Tilted
6	20–25	M	R	20/400	12	LP	Phakic	Trauma	Failed	Stable
7	80–85	M	L	CF	12	CF	Pseudophakic	BK, ACIOL	Clear	Stable
8	40–45	M	L	CF	13	20/200	Phakic	Trauma	Clear	Subluxated
9	80–85	M	R	CF	16	NLP	Pseudophakic	Graft failure, ACIOL	Failed	Stable
10	75–80	M	R	CF	18	LP	Phakic	Corneal scar	Failed	Stable
11	65–70	M	L	HM	10	HM	Aphakic	Corneal ulcer/scar	Clear	Stable
12	70–75	F	R	CF	23	20/400	Aphakic	BK	Failed	Stable
13	45–50	F	L	LP	24	CF	Phakic	Trauma	Failed	Stable
14	65–70	M	L	CF	20	HM	Pseudophakic	BK, dropped IOL	Failed	Stable
15	70–75	M	R	HM	10	HM	Pseudophakic	BK, ACIOL	Failed	Stable
16	60–65	F	R	HM	23	HM	Pseudophakic	BK, ACIOL	Failed	Stable
17	55–60	F	R	CF	14	20/60	Pseudophakic	Graft failure	Clear	Stable
18	20–25	M	L	CF	15	CF	Phakic	Trauma	Failed	Stable
19	60–65	M	L	20/160	22	20/200	Pseudophakic	Graft failure, ACIOL	Failed	Stable
20	75–80	M	L	CF	19	CF	Aphakic	BK	Failed	Stable
21	70–75	M	L	20/200	14	HM	Pseudophakic	Failed DSAEK, ACIOL	Failed	
22	50–55	M	L	20/200	21	20/125	Phakic	Trauma	Clear	Stable

VA: Visual acuity, IOP: Intraocular pressure, FU: Follow-up, M: Male, F: Female, R: Right, L: Left, CF: Counting fingers, HM: Hand motion, ACIOL: Anterior chamber IOL, BK: Bullous keratopathy

**Table 2:** Factors related to improvement of best corrected visual acuity by two or more lines at 12 months after combined PKP with sutured scleral fixated IOL

BCVA improvement by 2 or more lines		Improved (N = 13)		Not improved (N = 9)		Validation
		Number	%	Number	%	
**Main indicator**	Traumatic globe rupture	4	30.8	2	22.2	p = 0.3
	Bullous keratopathy.	5	38.5	4	44.4	
	Failed graft.	4	30.8	1	11.1	
	Corneal scar.	0	0	2	22.2	
**Preoperative comorbidity**	Glaucoma	4	30.8	2	22.2	p = 0.5
	Vitreous hemorrhage	2	15.4	0	0	
	Retinal pathology	1	7.7	3	33.3	
	Other	1	7.7	1	11.1	
	None	5	38.4	3	33.3	
**Stable IOL at last follow-up**	Yes	11	84.6	8	88.9	RR = 0.95, 95% CI 0.7; 1.3 p = 0.8
	No	2	15.4	1	11.1	
**Graft status after surgery**	Clear	6	46.2	3	33.3	RR = 1.4, 95% CI 0.5; 4.1 p = 0.6
	Not clear	7	53.8	6	66.7	

## Discussion

In a tertiary eye hospital in Eastern Saudi Arabia, complex eye conditions were treated with a triple procedure: PKP, management of the lens or existing IOL, and insertion of a scleral fixed IOL. Ophthalmologists often perform complex and challenging surgeries on eyes with corneal pathologies and unstable or weak posterior capsules that require IOL fixation during implantation. In countries such as Saudi Arabia, where local donations do not exist yet, imported corneas require judicious use. The results of the present study are encouraging, showing improved vision in more than half of the eyes, with no cases requiring IOL removal due to severe complications.

Similar to a large series of 96 cases involving patients who underwent scleral-fixated IOL implantation combined with PKP published in Germany [15]. The incidence rate of bullous keratopathy is 1–2% among eyes operated for cataracts [16]. With a rapid increase in the number of cataract surgeries in industrialized countries like Saudi Arabia and with new trainees gaining practical experience during residency training at our institute, iatrogenic endothelial damage is possible, as reflected by the one-third of the eyes with bullous keratopathy included in our cohort.

Improved vision is the main objective of ophthalmic intervention, and our cohort showed 50–60% success in this regard by avoiding blindness and improving the distance vision by at least two lines. To our knowledge, no large-scale study has been conducted in recent years using modern technology and scleral-fixated IOL, with which we can compare our outcomes. However, Jonas et al. reported on 135 patients who underwent PKP with IOL implantation in the posterior chamber [17]. Those who underwent sutured scleral-fixated IOL implantation combined with PKP were 23 out of 135 eyes. The mean visual acuity improvement in this subgroup was one line. Their profile and preoperative ocular status were comparable to those in our study, with trauma and bullous keratopathy being the main indications for surgery in this subgroup.

Various methods stabilize the lens in the posterior chamber, including scleral fixation of the haptic using sutures in the scleral tunnel, tied sutures, sutures to the iris, and glue. Stability of the IOL optic is crucial for long-term success [6, 7, 18]. In our study, 3 (13.6%) eyes had unstable IOL at the last follow-up. There was no significant difference between eyes with stable IOLs and those with unstable IOLs. This may be explained by the presence of other comorbidities, such as advanced glaucoma and corneal graft failure, contributing to decreased vision in both groups. Moreover, Holland et al., in a series of 115 patients, did not report a single case of lens decentration [9]. Further review of the suture status in the scleral tunnel is required to evaluate the underlying cause of the improved IOL stability.

Our study’s graft survival rate was 41% at 3 years median duration. Reportedly, the graft survival rates at 1 year and 5 years after PKP to manage keratoconus were 99.8% and 97.6%, respectively [19]. The long duration required for surgery and additional manipulation required for scleral fixation of the IOL could have negatively affected the corneal viability. Moreover, the current study included patients who had presented preoperatively with traumatic globe injuries (27.3%), glaucoma (27.3%), and graft failure (22.7%), all of which are factors that may have lowered the graft survival rate. In another study which included a significant number of patients with glaucoma, peripheral anterior synechia and history of failed grafts, the secondary graft failure rate was as high as 55.55% [20]. Glaucoma and intraocular pressure increase can also develop postoperatively. In a US-based study, new onset worsening of intraocular pressure was observed in 31% of cases, and a similar rate was found in the present study [9].

Our study had a few limitations. The studied included a few patients which may limit the ability to perform inferential statistical tests and to draw specific conclusions. The retrospective nature of the study is also another limitation. With retrospective studies, incomplete documentation is another frequently encountered problem. Some of our patients had additional ocular comorbidities such as vitreous hemorrhage, retinal detachment, and advanced glaucoma. Therefore, preoperative and postoperative vision may not accurately reflect the status of the cornea and IOL. Furthermore, optical coherence tomography of the macula was not part of the routine postoperative workup and would provide more insight in cases with a clear view.

## Conclusion

The combination of sutured scleral-fixated intraocular lens implants and PKP is an effective intervention for preserving visual acuity in patients with complex cases. However,  graft survival and the need for repeat corneal transplantation should be taken into consideration when evaluating patients for combined procedures.

## Data Availability

The datasets used and/or analyzed during the current study are available from the corresponding author on reasonable request.

## Abbreviations

IOL: Intraocular lens
PKP: Penetrating keratoplasty
BCVA: Best corrected visual acuity
PCIOL: Posterior chamber intraocular lens
